# Chitosan-Based High-Intensity Modification of the Biodegradable Substitutes for Cancellous Bone

**DOI:** 10.3390/jfb14080410

**Published:** 2023-08-03

**Authors:** Anna Kołakowska, Dorota Kołbuk, Andrzej Chwojnowski, Andrzej Rafalski, Agnieszka Gadomska-Gajadhur

**Affiliations:** 1 Faculty of Chemistry, Warsaw University of Technology, Noakowskiego St. 3, 00-664 Warsaw, Poland; anna.kolakowska.dokt@pw.edu.pl; 2 Institute of Fundamental Technological Research Polish Academy of Sciences, Pawińskiego St. 5B, 02-106 Warsaw, Poland; 3 Nalecz Institute of Biocybernetics and Biomedical Engineering, Polish Academy of Sciences, Ks. Trojdena St. 4, 02-109 Warsaw, Poland; 4 Radiosterilisation Facility for Medical Supplies and Transplants, Institute Nuclear Chemistry and Technology, Dorodna St. 16, 03-195 Warsaw, Poland

**Keywords:** chitosan, polylactide, bone substitute, tissue regeneration

## Abstract

An innovative approach to treating bone defects is using synthetic bone substitutes made of biomaterials. The proposed method to obtain polylactide scaffolds using the phase inversion technique with a freeze extraction variant enables the production of substitutes with morphology similar to cancellous bone (pore size 100–400 µm, open porosity 94%). The high absorbability of the implants will enable their use as platelet-rich plasma (PRP) carriers in future medical devices. Surface modification by dipping enabled the deposition of the hydrophilic chitosan (CS) layer, maintaining good bone tissue properties and high absorbability (850% dry weight). Introducing CS increases surface roughness and causes local changes in surface free energy, promoting bone cell adhesion. Through this research, we have developed a new and original method of low-temperature modification of PLA substitutes with chitosan. This method uses non-toxic reagents that do not cause changes in the structure of the PLA matrix. The obtained bone substitutes are characterised by exceptionally high hydrophilicity and morphology similar to spongy bone. In vitro studies were performed to analyse the effect of morphology and chitosan on cellular viability. Substitutes with properties similar to those of cancellous bone and which promote bone cell growth were obtained.

## 1. Introduction

Bone tissue can self-regenerate, but damages that are too large, so-called critical defects, cannot repair themselves [1]. The gold standard for treating such injuries is autologous grafts [2]. A transplant taken directly from the patient does not pose the risk of an undesirable immunological response. Nevertheless, it has disadvantages, including the formation of damage at the site of tissue extraction and the risk of secondary damage and deformities [3].

Synthetic bone substitutes made of biomaterials can also be used to treat critical defects, providing a scaffold on which bone cells, taken directly from the patient, can settle and proliferate [4,5]. The implants must be biocompatible, degrade to non-toxic products and have mechanical properties and morphology similar to bone tissue [6,7]. Cancellous bone substitutes should be porous (>80%); open porosity is preferred to exchange nutrients and cellular metabolic products and promote tissue vascularisation. The pore size, which significantly influences cell activity, should be greater than 100 µm, optimally 200–350 µm [8,9]. The absorbability of substitutes must be good to soak them in substances that enable and promote cell growth, e.g., platelet-rich plasma (PRP) that enhances bone grafts [10]. Bone substitutes are obtained using solution casting, phase separation, electrospinning or 3D printing [11]. The phase inversion technique with a freeze extraction variant enables the preparation of scaffolds with properties very similar to those of cancellous bone (patent no. PL P.425802 [12]).

Polylactide (PLA) is a non-toxic, biodegradable polyester readily used in tissue engineering. In the body, it degrades to lactic acid, a compound formed naturally during anaerobic muscle respiration [13]. The PLA degradation mechanism in in vitro conditions is surface. However, polylactide is a highly hydrophobic compound and does not promote cell adhesion [14]. Scaffolds can be modified with hydrophilic layers to improve surface properties [15]. The immersion method is simple and effective for modifying substitutes, which involves immersing the substitute in a modifying solution and then fixing the resulting layer (e.g., thermally) [16]. The modifying solution remains mainly on the surface of the scaffold; it can penetrate the substitute only through capillary forces. To increase the intensity of the process, the modification can be carried out in a vacuum. In the present work, we used a chitosan salt solution to modify the surface of polylactide [17]. Chitosan (CS) is a biocompatible, non-toxic polysaccharide, a deacetylated derivative of chitin. It is bioactive, degrades to non-toxic oligosaccharides and has osteoconductive properties [18]. CS is formed by D-glucosamine and N-acetyl-D-glucosamine units, with the former predominating. This makes the structure of CS similar to that of glycosaminoglycans found in the extracellular matrix [19]. The hydrophilic amino groups can undergo protonation, which gives chitosan the ability to form complexes with negatively charged molecules, e.g., lactic acid formed during the degradation of polylactide [20].

This study aimed to increase the hydrophilicity of polylactide bone substitutes via surface modification. The scaffolds were modified with chitosan, and their properties after the process were investigated. The morphology, mechanical properties, porosity and absorbability were changed after applying the CS layer. It was also found that the substitutes do not exhibit cytotoxicity, essential for their use as implants in treating bone damage.

## 2. Materials and Methods

Poly-L-lactide (PLLA) M_n_ of 86,000 g/mol and 2.20 dispersity (DI) (Nature Works, Plymouth, MN, USA) was used for obtaining scaffolds. Chitosan hydrochloride, deacetylation 85–95%, pharmaceutical grade (Golden Shell, Yuhuan, Zhejiang, China) and sodium hydrogen carbonate, analytical grade (POCH, Gliwice, Poland) were used for the modification process. 1,4-dioxane, 2-propanol and methanol, all analytical grade (POCH, Poland), and methanol of technical quality (BUTRA, Sopot, Poland) were used as organic solvents. Ultrapure water was obtained using a Mili-Q device (WUT, Warsaw, Poland). 

### 2.1. Preparation of Bone Substitutes [21]

A solution of PLLA in 1,4-dioxane was prepared using a PLLA/dioxane mass ratio of 0.03. Two samples were prepared, one without (PLA) and one with porphore (POR). For the POR type, the mixture was heated to 50 °C and the porophor (ultrapure water) was added with a porophor/solution ratio of 0.075. A total of 20 mL of prepared solution was poured into cylindrical polypropylene moulds and later frozen at −18 °C for 24 h. The frozen forms were removed from the moulds and put into a gelling bath (methanol) for five days at −18 °C. Subsequently, the substitutes were placed in a rinsing bath (distilled water) for 24 h at room temperature, and the rinsing bathwater was replaced twice. Substitutes were vacuum-dried for 24 h at 45 °C, and the pressure was under 10 mbar. The obtained substitutes were cylinders with a volume of about 10 cm^3^.

### 2.2. Surface Modification of Substitutes [12]

The chitosan hydrochloride (CS-HCl) solution was prepared by adding 4.5 g of CS-HCl to 150 mL of distilled water. The chitosan hydrochloride solution was heated to 30 °C and stirred at 600 rpm for 15 min. A substitute was placed in a vacuum ampoule, and 15 g of the solution was added. A weight was put on a scaffold. The ampoule was kept under vacuum in a water bath at 30 °C for 15 min. The substitute was frozen for 24 h at −18 °C and, after that time, for 72 h, with pressure below 0.10 mbar.

For POR samples, further treatment was implemented: a solution of 0.36% *w*/*v* sodium bicarbonate in water and methanol was prepared (water/methanol volume ratio of 1:1). The scaffold was placed in a vacuum ampoule, and 25 mL of NaHCO_3_ was added. The ampoule was kept under vacuum for 10 min. The substitute was placed in a rinsing bath (distilled water) at 37 °C for 3 h, and the rinsing bathwater was replaced twice. The scaffold was vacuum-dried for 48 h at 40 °C and 10 mbar pressure. 

### 2.3. Infrared Spectroscopy

Fourier transform infrared spectroscopy was analysed using Attenuated Total Reflection (FTIR-ATR). An ALPHA Bruker spectrometer (Warsaw, Poland) with an ATR attachment was used. Dry samples were tested without prior preparation. 

### 2.4. Elemental Analysis

The analysis used Elementar Vario EL III apparatus (Warsaw, Poland). The percentages of carbon, hydrogen and nitrogen in dry samples were determined. The expected content of elements was calculated based on open porosity measurements, assuming that chitosan with a deacetylation degree of 90% occupies the entire volume of open pores.

### 2.5. Scanning Electron Microscopy (SEM) [21]

The morphology of the substitutes was examined with a TM1000 Hitachi microscope (Poland). Cuboid fragments were removed from the substitutes and coated with a 7–9 nm thick gold layer in a K550X Sputter Coater. 

### 2.6. 3D Surface Modelling

Three-dimensional surface models of substitutes were prepared using a HIROX KH-7700 microscope with a 5040HS lens (Józefów, Poland).

### 2.7. Differential Scanning Calorimetry (DSC) 

Differential scanning calorimetry analysis was conducted in a Perkin Elmer Differential Scanning Calorimeter PYRIS-1 (Warsaw, Poland). Samples were heated from 0 to 250 °C with a temperature change of 10 °C/min.

### 2.8. Open Porosity (P_o_) and Mass Absorbability (A_m_) [22,23]

The substitutes were weighed with a Mettler Toledo XS 104 analytical scale (Warsaw, Poland). The samples were soaked in isopropanol (measuring medium) under vacuum conditions for 30 min. The open porosity and bulk absorbability of samples were determined from formulae based on Archimedes’ law:

(1) $$P_{o}=\frac{m_{\mathrm{np}}-m_{s}}{m_{\mathrm{np}}-m_{n}}\cdot 100\%$$

(2) $$A_{m}=\frac{m_{\mathrm{np}}-m_{s}}{m_{s}}\cdot 100\%$$

where m_s_—dry sample mass, m_np_—the mass of the sample soaked in the measuring medium and air-weighed and m_n_—the mass of the sample soaked in measuring medium and weighed in liquid. Three samples of each type were measured, the result was averaged and the standard deviation was reported.

### 2.9. Elasticity

Cylinders 6–8 mm high were cut from the centres of the bone substitutes. Static compression tests were performed on Tinius Olsen H104S apparatus (Warsaw, Poland) using QMat 5M5 software at a compression rate of 10% of the cylinder height per minute (ISO 844). Young’s modulus was calculated as a tangent modulus of a straight-line section of the stress–deformation curve (the deformation ranges up to 15%).

### 2.10. Surface Free Energy Determination [24]

Contact angle and surface free energy were measured for two liquids: diiodomethane and water. A drop of liquid was applied to the scaffold surface. A photograph of the sessile droplet on the surface was then taken. In the program ToupView (Poland), the angle between the surface and the drop of the measuring liquid was determined by drawing a tangent. Using the Owens–Wendt model, the surface free energy (SFE) of the scaffold was calculated as a sum of dispersion and polar components according to the following formula:

(3) $$\gamma_{S}=\gamma_{S}^{p}+\gamma_{S}^{d}$$

where $\gamma_{S}$ is the substitute surface free energy, $\gamma_{S}^{p}$ is the polar component and $\gamma_{S}^{d}$ is the dispersion component of the surface free energy of the substitute.

The dispersion and polar component values of surface free energy were calculated according to the formulae:

(4) $${\left(\gamma_{S}^{d}\right)}^{1/2}=\frac{\gamma_{\dim}\left(\cos\theta_{\dim}+1\right)-\gamma_{w}\left(\cos\theta_{w}+1\right)\sqrt{\frac{\gamma_{\dim}^{p}}{\gamma_{w}^{p}}}}{2(\sqrt{\gamma_{\dim}^{d}}-\sqrt{\frac{\gamma_{\dim}^{p}\gamma_{w}^{d}}{\gamma_{w}^{p}}})}$$

(5) $${\left(\gamma_{S}^{p}\right)}^{1/2}=\frac{\gamma_{w}\left(\cos\theta_{w}+1\right)-2\sqrt{\gamma_{S}^{d}\gamma_{w}^{d}}}{2\sqrt{\gamma_{w}^{p}}}$$

where:

$\gamma_{\dim}$—the SFE of diiodomethane (50.80 mJ/m^2^);
$\gamma_{\dim}^{p}$—the polar component of diiodomethane SFE (0.00 mJ/m^2^);
$\gamma_{\dim}^{d}$—the dispersive component of diiodomethane SFE (50.80 mJ/m^2^);
$\theta_{\dim}$—the measured contact angle of diiodomethane;
$\gamma_{w}$—the SFE of water (72.80 mJ/m^2^):
$\gamma_{w}^{p}$—the polar component of water SFE (51.00 mJ/m^2^);
$\gamma_{w}^{d}$—the dispersive component of water SFE (21.80 mJ/m^2^);
$\theta_{w}$—the measured contact angle of water.

### 2.11. Cytotoxicity Analysis [21]

Sample sterilisation: All samples were sterilised with high-energy electrons (electron energy: 9.1 MeV; dose 15.0 kGy).

Cubic sections of substitutes with dimensions of 2 mm × 2 mm × 2 mm were prepared for the tests.

Fibroblasts culture: In vitro tests were carried out using the MG63 line of fibroblasts (ATTC Sigma Aldrich, Poznan, Poland). Cells were cultivated in 25 cm^3^ flasks containing a medium of high-glucose Dulbecco’s modified Eagle’s medium (DMEM, Taufkirchen, Germany), 10% foetal bovine serum (FBS, Taufkirchen, Germany), 1% glutamine and 1% antibiotics. Cells were incubated in a 5% CO_2_ environment at a temperature of 37 °C. Harvesting of the cells took place in 70–80% confluent flasks. In the first step, cells were washed twice in phosphate-buffered saline (PBS, Taufkirchen, Germany). After that step, 5 mL of 0.05% of trypsin solution was added to the cells and they were placed in the incubator for a few minutes. Then, the flask was tapped delicately to detach the cells. After obtaining the harvested cells, 10 mL of culture medium was added and centrifuged. The centrifugation was carried out in ambient temperature conditions at 170× *g* for 5 min. The required cell density was obtained due to the pellet being resuspended with a culture medium and then diluted.

PrestoBlue test: To obtain the cytotoxicity, 4 samples of each sample type were placed in a 48-well plate. The MG63 cell suspension was seeded on them with a density 3 × 10^4^ cells/well and the samples put in an incubator for 1 and 3 days. After that, the medium was removed. Each well was filled with 90 μL of PBS and 10 μL of PrestoBlue reagent. Then, the plate was returned to the incubator for 45 min. When this step was completed, 100 μL of the solution from each well was transferred to a 96-well plate. The fluorescence read with excitation/emission 530/620 nm filters was measured using 530/620 nm excitation/emission wavelengths in Fluoroskan Ascent FL Thermo Fisher Scientific. Data were compared using a one-way ANOVA test. The data were considered significantly different when *p* < 0.05. In the case of in vitro analysis, the differences between the groups were tested for their statistical significance using a non-parametric two-tailed T-test using GraphPad Prism. A *p*-value of less than 0.05 was considered statistically significant. 

## 3. Results

### 3.1. Preparation of Substitutes

**Figure 1:**
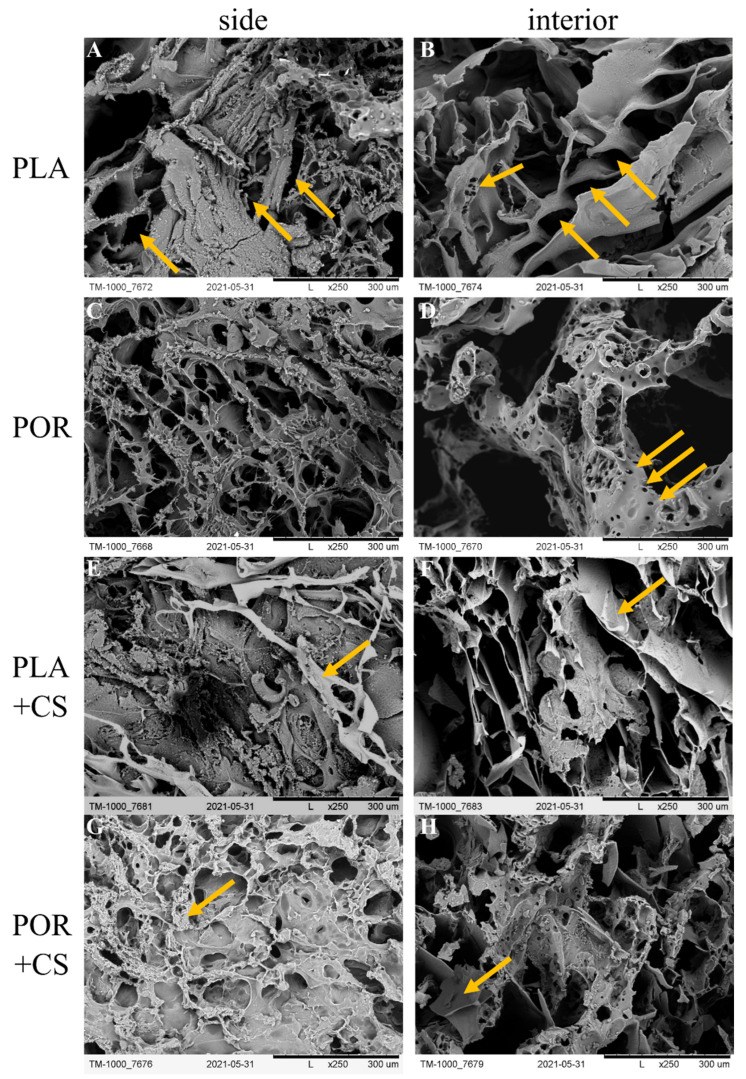
SEM images of PLA, POR, PLA + CS and POR + CS bone substitutes. (**A**). side of PLA scaffold without porophor, (**B**). interior of PLA scaffold without porophor, (**C**). side of PLA scaffold with porophor, (**D**). interior of PLA scaffold with porophor, (**E**). side of PLA and chitosan scaffold without porophor, (**F**). interior of PLA and chitosan scaffold without porophor, (**G**). side of PLA and chitosan scaffold with porophor, (**H**). interior of PLA and chitosan scaffold with porophor.

**Figure 2:**
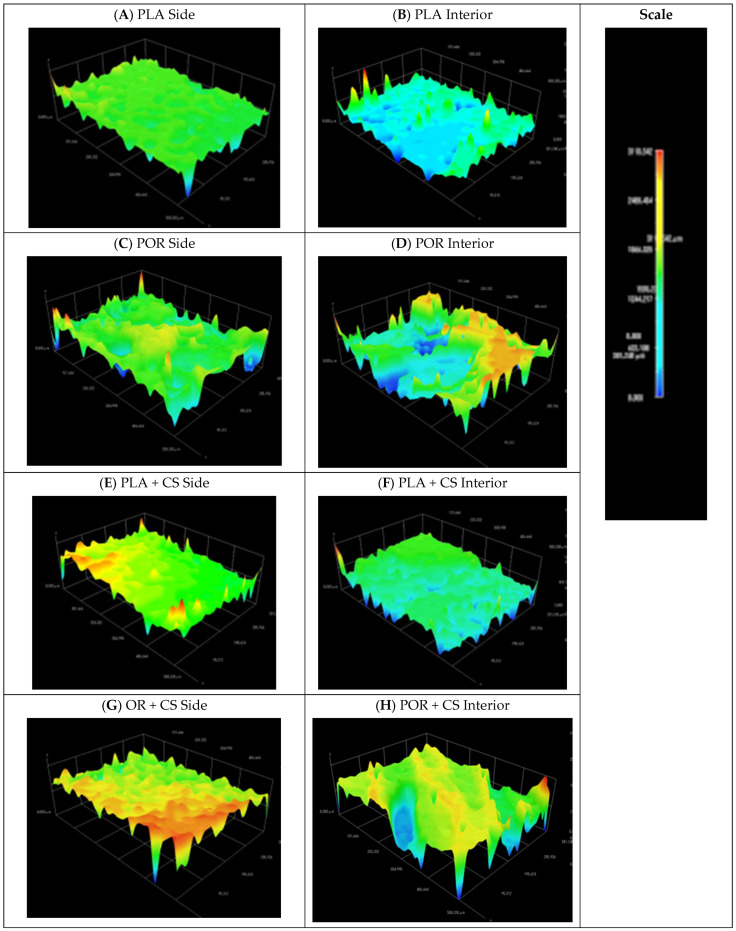
Models of side and interior parts of PLA, POR, PLA + CS and POR + CS scaffolds. (**A**). side of PLA scaffold without porophor, (**B**). interior of PLA scaffold without porophor, (**C**). side of PLA scaffold with porophor, (**D**). interior of PLA scaffold with porophor, (**E**). side of PLA and chitosan scaffold without porophor, (**F**). interior of PLA and chitosan scaffold without porophor, (**G**). side of PLA and chitosan scaffold with porophor, (**H**). interior of PLA and chitosan scaffold with porophor.

Two cylindrical substitutes were obtained using the phase inversion method with the freeze extraction variant: the PLA samples were prepared without and the POR with microporophor. The scaffolds’ morphology was determined using the SEM technique (Figure 1), and 3D models of the inner and lateral surfaces of the substitutes were made (Figure 2).

The two types of scaffolds differed in morphology. The outer surface of the PLA substitute made without porophor (Figure 1A) was characterised by pores of irregular shapes: oval or elongated, with sizes of 50–250 µm. The pores were distributed unevenly, with pore-free zones. The side of the POR substitute (Figure 1C) was characterised by oval pores of 100–400 µm occurring throughout the surface. Flocs of short-chain polylactide were visible. The interior of the PLA scaffold (Figure 1B) was also characterised by pores of irregular, elongated shapes of 100–400 µm, arranged in a herringbone pattern. Small local zones of micro-perforation were present. The interior of POR (Figure 1D) had more regular, oval pores (200–700 µm) with microperforated walls (tens of micrometres in diameter).

From 3D models of the scaffold surfaces, the difference in the roughness of the systems depending on the preparation method can be seen. The systems without porophor (Figure 2A,B) were more flattened and showed less surface diversity than those made with porophor (Figure 2C,D). The outer layers of both substitutes were more flattened than the interiors, which can be attributed to touching the walls of the moulds in the production process. On the lateral surface of the POR, spherical pores could be distinguished.

### 3.2. Surface Modification

Chitosan chloride was successfully deposited on the prepared substitutes through a dip coating method (+CS substitutes). Chitosan was deposited mainly on the outer layers of the substitutes (Figure 1E,G), forming elaborate systems. In the PLA + CS scaffolds, the polysaccharide resembles a lace woven from a single thread, whereas in POR + CS, the chitosan is visible as non-porous patches covered by a lace-like arrangement on top. Much less chitosan could be seen in the interior of the scaffolds (Figure 1F,H) than on the lateral surface; the polysaccharide did not form extensive systems but was visible locally as small, non-porous patches.

Spatial models of scaffolds sides (Figure 2E,G) showed increased roughness of the surfaces after the chitosan layer deposition. There was no significant effect of applying chitosan layers on the models of the substitutes’ internal surfaces.

**Figure 3:**
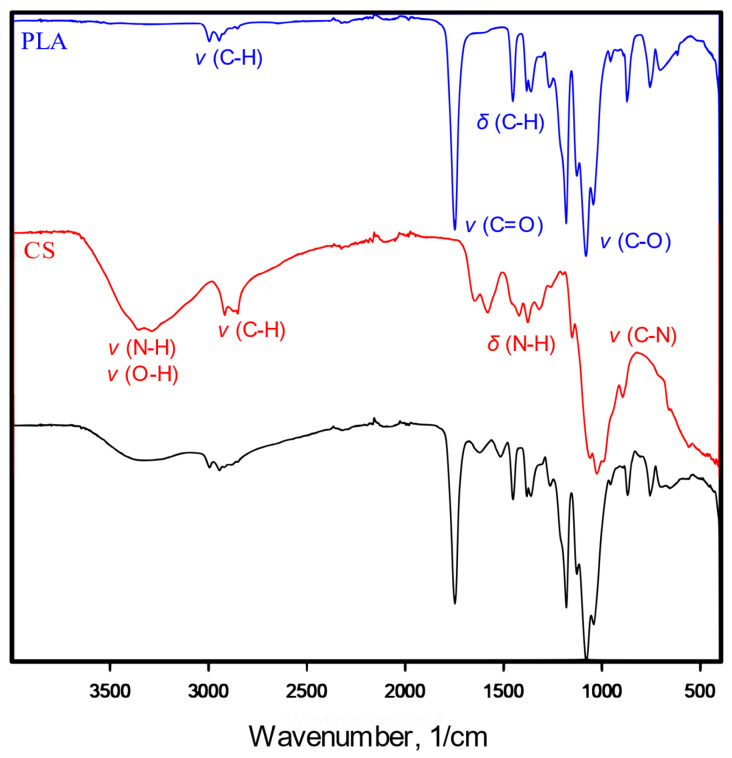
IR spectrum of pure poly-L-lactide bone substitutes (PLA), chitosan (CS) and chitosan-modified substitutes (PLA + CS).

In the IR spectrum (Figure 3) of the chitosan-modified scaffold, characteristic spectra of both polylactide and chitosan were found. Stretching vibrations of C–H (3000 cm^−1^), C=O (1747 cm^−1^) and C–O (1180–1042 cm^−1^) and deforming vibrations of C–H (1360 cm^−1^) are characteristic of polylactide, as shown in the PLA spectrum. The presence of chitosan (shown as CS spectrum) in the POR + CS scaffolds is confirmed in stretching vibrations of O–H and N–H bonds (3400–3200 cm^−1^) and deforming vibrations of N–H (1646–1580 cm^−1^) and O–H (1420–1320 cm^−1^).

The unmodified scaffolds are made up exclusively by the elements present in the polylactide: carbon, hydrogen and oxygen (Table 1).

The percentage distribution of each component of the test sample matches the content calculated from the chemical formula of the polylactide. Chitosan-modified scaffolds are enriched with nitrogen derived from the amino groups of chitosan. The expected chitosan content was calculated based on the open porosity of the substitutes—the values given in Table 1 correspond to the complete filling of the open pores by chitosan. The calculated nitrogen content of PLA and POR is the same, i.e., 8.5%. The experimental results are lower, around 3%. The difference may be due to the mechanical blocking of access to the interior of the substitutes by the viscous solution of the chitosan salt or the leaching of small amounts of CS during the polysaccharide precipitation process.

### 3.3. Properties of Substitutes

**Figure 4:**
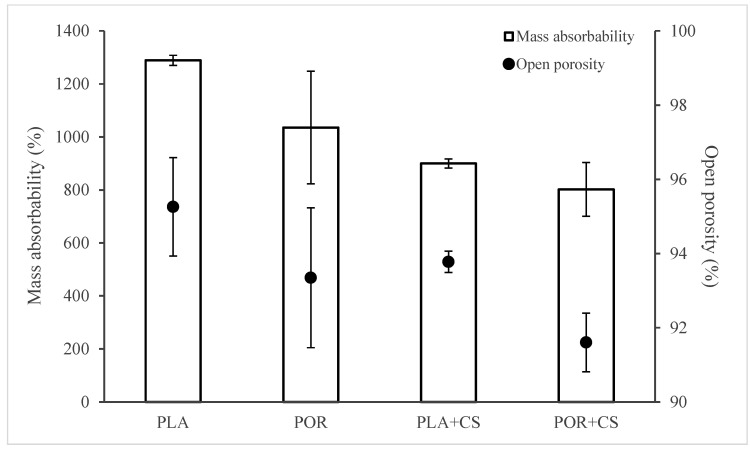
Mass absorbability and open porosity of PLA, POR, PLA + CS and POR + CS substitutes.

The substitutes’ open porosity and mass absorbability were determined based on hydrostatic weighing in isopropanol (Figure 4). Water cannot be used as a measuring medium because of the poor wettability of the polylactide, which is not the case for isopropanol. Using two measuring media would make it impossible to compare results; therefore, for all samples, excellent surface-wetting alcohol was used. The PLA scaffolds have the highest absorbability of 1300% of the weight of the dry substitute. The addition of porophor causes a decrease in the value to 1100% and an increase in the heterogeneity of the systems, as seen in the rise in the standard deviation. When modified with chitosan, the absorbability of scaffolds decreases, regardless of the preparation method. This is due to the partial pore sealing with polysaccharide settling. Also, after modification, scaffolds without porophor are characterised by higher absorbability than the POR type (900% and 800%, respectively).

The trend of changes in open porosity values of the scaffolds is similar to the changes in mass absorbability (Figure 4). Applying microporophor or surface modification reduces the open porosity of the substitute. The PLA scaffold has the highest porosity (approximately 95%), and POR + CS has the lowest porosity (approximately 91%). The remaining substitutes are characterised by similar porosity of about 93%.

Using microporophor and modification with chitosan decreases the scaffolds’ mass absorbability and open porosity.

**Figure 5:**
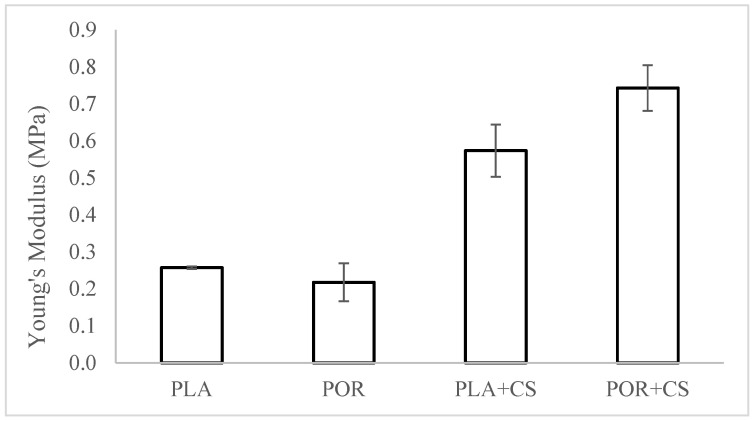
The elasticity of PLA, POR, PLA + CS and POR + CS substitutes.

The mechanical properties of both types of substitutes were evaluated before and after modification (Figure 5). The static compression technique was used, and samples for the tests were cut out from the middle part of the scaffold. The values of Young’s modulus were determined based on a linear part of the function on the diagram of dependence of tension on the implant’s relative deformation. The Young’s moduli of the PLA and POR substitutes are similar, about 0.25 MPa. The modified PLA + CS scaffolds have a modulus of about 0.60 MPa, while that of POR + CS is 0.75 MPa. After modification with chitosan, the value of Young’s modulus increases; that is, the stiffness of the substitute increases. This is due to applying a layer of rigid chitosan and reducing the available space in the system.

**Figure 6:**
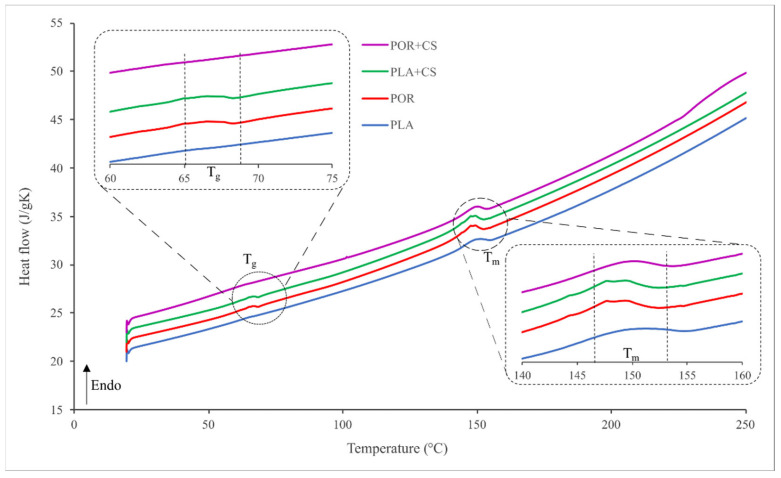
Thermograms of side and interior parts of PLA, POR, PLA + CS and POR + CS substitutes.

The thermal properties of the substitutes were investigated using the differential scanning calorimetry technique. Thermograms for PLA, POR, PLA + CS and POR + CS systems are similar (Figure 6). Two endothermic transitions can be seen: 65–70 °C is the glass transition temperature of polylactide and 145–155 °C is the melting temperature. No effect of porophor or chitosan on thermal properties is observed.

**Figure 7:**
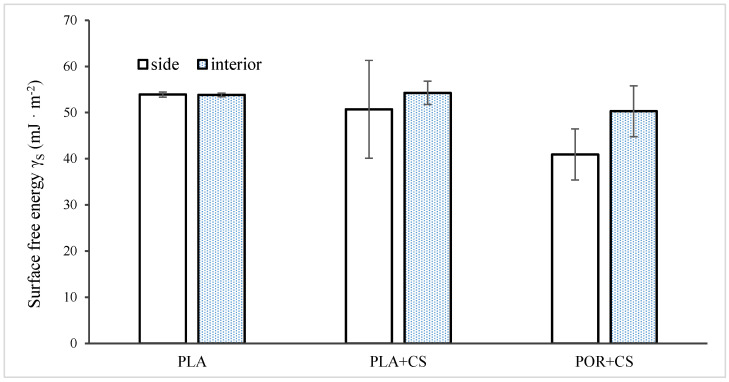
Surface free energy of side and interior parts of unmodified and modified substitutes.

The wetting angle of the substitutes was examined for the external and internal surfaces of the substitutes (Figure 7). The surface free energy (SFE) value calculated according to the OW model for PLA is about 54 mJ/m^2^. Modified scaffolds differ in their properties on the interior and exterior walls. The sides of the scaffolds are characterised by lower mean values (50 mJ/m^2^ for PLA + CS, 40 mJ/m^2^ for POR + CS) with high standard deviations, which indicates the high heterogeneity of these surfaces. The SFE values of the PLA + CS and POR + CS interiors are similar to the SFE of the unmodified PLA scaffold. Nevertheless, the deviations are more significant, suggesting that chitosan enters the interiors of the substitutes to a small extent.

**Figure 8:**
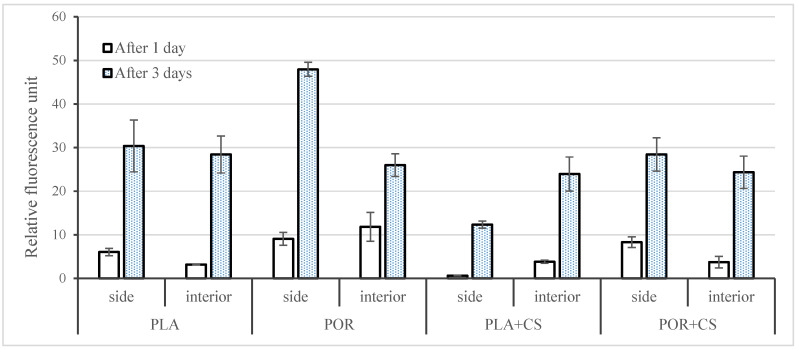
Cell viability of MG 63 cells cultured on side and interior parts of PLA, POR, PLA + CS and POR + CS samples for 1 and 3 days post-seeding. Error bars represent the standard deviation of the mean. Results were considered significant when *p* < 0.05.

Cell viability was analysed after 1 and 3 days (Figure 8). PLA, as a medical polymer, is non-cytotoxic. Additionally, porosity enhances the viability of cells seeded on PLA (POR sample). CS added to the PLA sample (PLA + CS) reduces viability, especially in the case of the PLA + CS side. This effect was not observed in POR + CS, indicating similar cellular viability to PLA.

## 4. Discussion

The three-dimensional scaffolds obtained via the phase inversion method with the freeze extraction variant without porophor (PLA) are characterised by pores of varying sizes and shapes [24]. There were also differences in the appearance of the pores depending on the surface area tested, as noted in other reports [25].

The method we developed is a low-temperature method of changing the surface of implants made of PLA. The properties of PLA itself do not change thanks to the use of low temperatures. The degree of crystallinity and molar mass does not change, contrary to what was previously reported by other authors. The morphology and pore size remain unchanged, distinguishing our method from previously described methods.

Introducing the porophor (POR samples) affects the scaffold’s morphology, producing pores of more regular shapes and increasing their interconnectivity and roughness of surfaces [26,27]. The pores in both systems are 100–400 µm and optimal for bone regeneration [28]. Micro-perforations in POR substitutes are an excellent advantage of substitutes as micropores support the processes of gas and nutrient exchange [29,30]; therefore, implants produced with microporophor are better suited for bone regeneration. The mass absorption of both substitute types exceeds 1100% of their weight. This trait will allow saturating the scaffolds with the patient’s platelet-rich plasma, stem cells or blood. As a target application, the implant will be a PRP carrier to aid bone regeneration [31,32,33]. The open porosity of the substitutes is high (about 94%), similar to that of cancellous bone [34]. The Young’s moduli of scaffolds (0.25 MPa) are lower than average values for cancellous bone (0.1–2 GPa [35]), meaning the substitutes are more flexible than bone. The substitutes were not designed to carry mechanical loads, and the high elasticity may facilitate implant insertion. The discrepancy in the Young’s moduli of spongy bone and scaffolds should not be considered a disadvantage. The value of the SFE calculated according to the OW model for polylactide is about 54 mJ/m^2^, which is higher than the literature values [36,37]. The difference is likely due to the roughness of the tested surface caused by the high porosity of the samples.

The scaffolds were modified by using the immersion method. A reduced pressure was applied to increase the intensity of the soaking process and facilitate the penetration of the solution into the substitutes, similar to [38]. For samples without porophor (PLA + CS), chitosan hydrochloride layers were left on, while additional chitosan precipitation was carried out for those with microporophor (POR + CS). Salt precipitation of chitosan removes unwanted chloride ions and reduces the risk of acidification of the implant environment. In the SEM images, chitosan is mainly visible on the outer surfaces and is reluctant to penetrate the interior of the scaffolds. It may be related to mechanical blocking of access due to the high viscosity of the solution or the smaller pore size of the scaffold’s outer surfaces. Introducing chitosan also increases surface roughness outside and inside, promoting cell adhesion [16].

FTIR analysis showed the presence of vibration bands characteristic for polylactide and chitosan in the modified substitutes. Nitrogen comprised about 3% of the samples tested, while the calculated maximum nitrogen content was 8.5%. This again confirmed that the modifying solution is reluctant to penetrate the substitutes. Note that the calculation is subject to error because it does not consider that chitosan settles primarily on the outer surface of the substitute, as stated previously. Both mass absorbability and open porosity decrease after the modification (from about 1100% and 94% to about 850% and 93%, respectively). This due to the reduction in available space. The presence of CS patches may impede fluid permeation into the substitute, but the scaffold can still absorb more than eight times its dry weight and be used as a carrier for platelet-rich plasma. After modification with chitosan, the value of the Young’s modulus increases almost threefold to about 0.7 MPa. As mentioned earlier, this value is lower than the Young’s modulus of spongy bone, but this does not affect the intended use of substitutes. The increase in stiffness is due to applying a layer of rigid chitosan and reducing the available space in the system—the air in the pores increases the elasticity of the scaffolds. No effect of porophor or chitosan introduction on thermal properties is observed. Introducing CS alters the scaffolds’ surface free energy and increases the surfaces’ inhomogeneity. Changes are mainly observed on the sides of the scaffolds, where most CS settles (decrease in SFE from 54 to approx. 45 mJ/m^2^ with multiple increases in the standard deviation). The SFEs of scaffolds’ interiors remain similar before and after modification; there is a less intense increase in surface diversity (increase in standard deviation), which is consistent with the problem of polysaccharide penetration into the interior.

Cellular viability, proliferation and migration depend on substrate properties related to the scaffold’s formation technology and the reagent chemistry [39,40]. Pore size, roughness and mechanical and surface properties may enhance the growth of osteoblasts [41,42]. After 1 day, when the surface properties determined protein adsorption and cellular behaviour, the highest cellular viability was observed on POR. Micropores and mechanical properties similar to PLA characterise this scaffold. Adding CS to PLA and POR reduced mass absorbability and total porosity—these parameters influenced the viability of the PLA + CS side, despite the bioactive nature of the chitosan. The decreased surface free energy combined with microporosity characteristic for POR + CS reduces the negative effect of other parameters. Microporosity enhances the specific surface area, roughness and related protein adsorption. Proteins may more easily attach to the surface and stimulate the osteogenic-related functions of cells, such as attachment, proliferation, osteogenic differentiation and biomineralisation [43]. Additionally, microporosity suitable for the POR and POR + CS scaffolds enhances the transport of biofactors, nutrients and metabolic waste between the scaffold and the environment [44].

## 5. Conclusions

The obtained substitutes are characterised by morphology and porosity similar to those of spongy bone. The shape and size of the pores depend on the preparation technique; better results were obtained in the production with microporophor. Surface modification with chitosan increases the heterogeneity of the systems and creates areas of increased hydrophilicity. Chitosan settles on the surface in threads, locally increasing the hydrophilic character, and is reluctant to penetrate the scaffolds. Introducing chitosan into the substitutes increases the roughness of their surface, without significantly affecting their porosity. The bulk absorbability of the substitutes after modification is high, allowing plasma to soak into the implants. The stiffness of substitutes after modification also increases.

## Figures and Tables

**Table 1:** Content of carbon, hydrogen, oxygen and nitrogen in polylactide substitutes; experimental and calculated based on the chemical structure of polylactide.

	Experimental Element Content (%)				Calculated Element Content (%)			
	C	H	O	N	C	H	O	N
PLA	50.1	5.6	44.3	0.0	50.0	5.6	44.4	0.0
PLA + CS	45.8	5.8	45.4	3.0	45.1	6.5	39.9	8.5
POR + CS	47.8	5.8	43.7	2.7	45.1	6.5	39.9	8.5

## Data Availability

The data presented in this study are available on request from the corresponding author.
